# Impact of terminologies for tumor pathology structured reports

**DOI:** 10.1186/1746-1596-8-S1-S22

**Published:** 2013-09-30

**Authors:** G Haroske, T Schrader

**Affiliations:** 1 Institute of Pathology, Dresden-Friedrichstadt General Hospital, Dresden, Germany; 2 Department Informatics and Media, University of Applied Sciences Brandenburg, Germany

## Background

For information exchange and data mining structured reports in tumor pathology have to be based on controlled vocabulary as to get a model of meaning. SNOMED CT, originated from a pathology terminology in the U.S. and Great Britain will probably become a global reference health terminology. With SNOMED CT or UMLS very comprehensive medical terminologies exist, which are reference terminologies for tumor pathology applications, too.

However, their direct use for structured reporting is limited so far, because many pathologists are rather bound to data entry systems agreed regionally or nationally and based on pre-digital recommendations and templates, published by scientific boards. As to fill the gap between well-known, often inconsistent and poorly agreed documentation tools and the comprehensive reference terminologies, interface terminologies are being developed [1,2].

National and international initiatives are necessary to reach a growing agreement on particular aspects and needs towards it. Interface terminologies might be a tool for drawing existing separate terminology systems to a finally global standard.

## Methods

Controlled vocabularies in guidelines of German pathologists for colorectal, breast and prostate cancer [3], in the basic tumor documentation of German cancer registries [4], and in the German HL7 Diagnoses Implementation Guide (http://wiki.hl7.de/index.php/IG:HL7_diagnosis), all standing for German terminologies in the field of tumors, have been mapped to PathLex, an interface terminology developed by IHE [5]. For all German terms without successful PathLex mapping a mapping to LOINC and SNOMED CT was performed. The mapping was done by comparing the German concepts term by term with the PathLex terms and the UMLS concepts using the terminology server http://terminology.vetmed.vt.edu/sct/menu.cfm for SNOMED CT and the current LOINC database http://search.loinc.org/.

## Results and discussion

**Figure 1:**
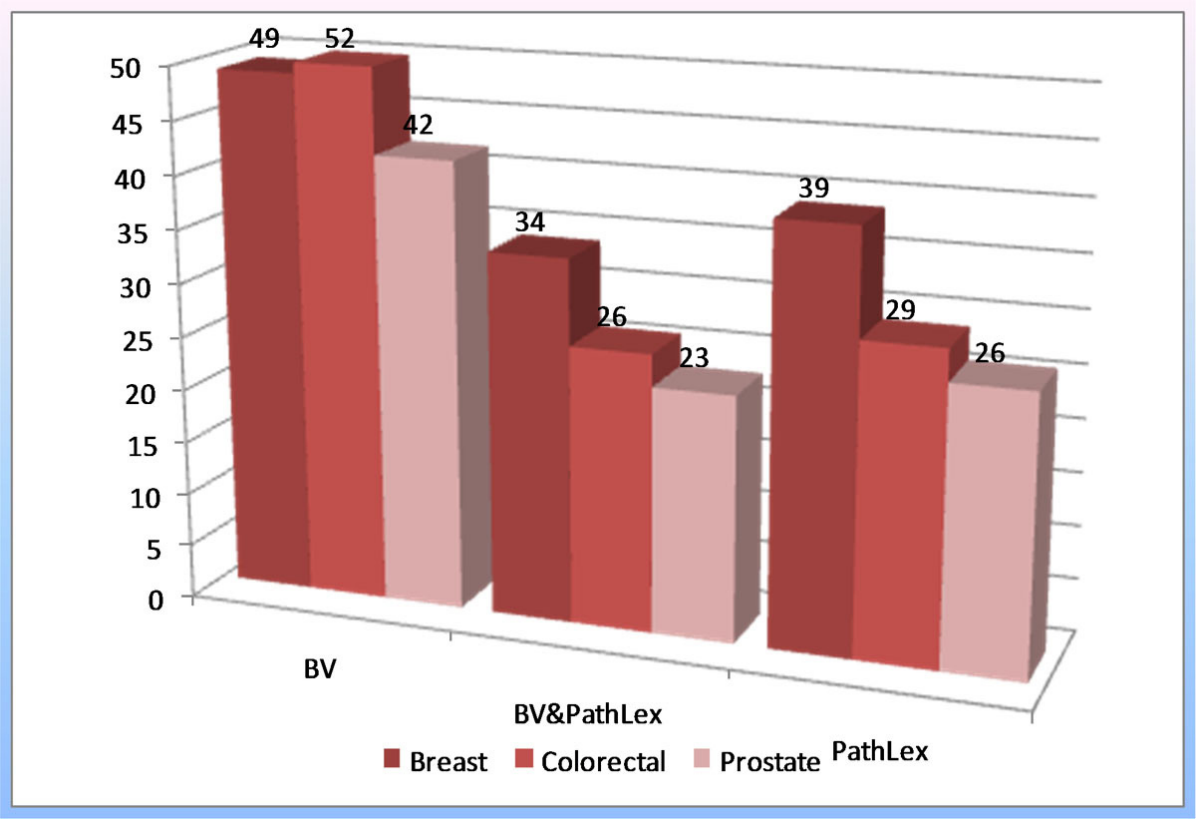
Number of terms in German Pathology Guidelines (BV), PathLex and commonly occurring in BV and PathLex for organspecific structured reports

On average a pathology guideline describes 40 to 50 terms which have to be registered as to fulfill the minimum documentation requirements. PathLex provides between 30 to 40 terms per tumor entity, less than 80% of them exactly match the German guideline vocabulary (Figure 1). Only a few German terms could be split in components, then matching PathLex terms. The coincidence of PathLex with HL7 Germany vocabulary or the basic data set of cancer registries is still lower, because these are rather classifications than terminologies and are focused on TNM and ICD. Furthermore the German guideline vocabulary, although organ oriented, does not differentiate between generic and organ specific information. This leads to imperfect mapping, too.

The remaining gaps could also not completely be filled with LOINC codes or SNOMED CT terms. There still exist very special terms used in the national scope only.

So far there is no standard on structured reporting in tumor pathology in Germany. The appropriate guidelines having been agreed during the last five years are based on a mixture of national and international terminologies and classifications, as those are used in the daily routine of pathologists, registrars and clinicians. With the upcoming use of HL7 CDA pathologists, too, are interested to use checklists and templates for structured reporting.

The mapping of national terminologies (which are interface terminologies, too) to an internationally agreed interface terminology and to reference terminologies clearly showed that some essentials of terminology building have to be given: clearly and uniquely defined medical concepts, defined terminology structure, relationships and codes. All of them are more or less lacking in one or the other terminology studied. Furthermore, terminologies, nomenclatures and classifications should more clearly divided from each other [6]. Mixing up classifications with terminology is causing some problems with life cycles of those terminologies. Life cycle management of interface terminology should be agreed as soon as possible.

## Conclusions

Although based on internationally agreed understanding, sharing the same concepts of tumor pathology, the terminology differences among the different sources are quite obvious. Those differences have to be overcome as to ascertain a reliable information exchange between different actors in the care of tumor patients. Increased attention should be paid to the focus of terminology building: basic observable entities for anatomic pathology reports, such as staining characteristics (intensity, pattern, structures stained) evaluation of immunohistochemical reactions, molecular tests, etc. should be included in the scope of activities.

Terminology mapping is one solution, but not the optimal one. Due to the ontological properties of reference terminologies an ontological approach seems to be successful and should therefore be taken into consideration.

The toolbox for terminology development has to be improved. In all steps of mapping a link to the concepts underlying the terms studied must be available. A closer collaboration with international terminology bodies as well as a sharpened realization of the impact of terminology in home made guidelines and beginning with ontology construction would contribute to an advanced progress of structured reporting in routine.

## List of abbreviations

SNOMED CT: Systematized Nomenclature of Medicine Clinical Terms; HL7: Health Level 7; IHE: Integrating the Healthcare Enterprises; LOINC: Logical Observation Identifiers Names and Codes; UMLS: Unified Medical Language System

## Competing interests

The authors declare that they have no competing interests.
